# Impairment of entorhinal cortex network activity in Alzheimer’s disease

**DOI:** 10.3389/fnagi.2024.1402573

**Published:** 2024-05-31

**Authors:** Farnaz Karimani, Afsaneh Asgari Taei, Mohammad-Reza Abolghasemi-Dehaghani, Mir-Shahram Safari, Leila Dargahi

**Affiliations:** ^1^Neuroscience Research Center, Shahid Beheshti University of Medical Sciences, Tehran, Iran; ^2^School of Electrical and Computer Engineering, College of Engineering, University of Tehran, Tehran, Iran; ^3^Neurobiology Research Center, Shahid Beheshti University of Medical Sciences, Tehran, Iran

**Keywords:** Alzheimer’s disease, entorhinal cortex, network activity, neuronal excitability, neural oscillations

## Abstract

The entorhinal cortex (EC) stands out as a critical brain region affected in the early phases of Alzheimer’s disease (AD), with some of the disease’s pathological processes originating from this area, making it one of the most crucial brain regions in AD. Recent research highlights disruptions in the brain’s network activity, characterized by heightened excitability and irregular oscillations, may contribute to cognitive impairment. These disruptions are proposed not only as potential therapeutic targets but also as early biomarkers for AD. In this paper, we will begin with a review of the anatomy and function of EC, highlighting its selective vulnerability in AD. Subsequently, we will discuss the disruption of EC network activity, exploring changes in excitability and neuronal oscillations in this region during AD and hypothesize that, considering the advancements in neuromodulation techniques, addressing the disturbances in the network activity of the EC could offer fresh insights for both the diagnosis and treatment of AD.

## 1 Introduction

Alzheimer’s disease (AD) is a progressive neurodegenerative disorder that impairs cognitive function in older people. The search for an effective treatment for this disease and the identification of its exact pathology are still among the most important concerns of medical research due to the global aging of the population and its economic and social impact (Alzheimer’s Disease, 2021). Amyloid-beta (Aβ) plaques and neurofibrillary tangles (NFTs) of tau are central to the pathogenesis of AD (Knopman et al., 2021). Other hypotheses in this area include the cholinergic neuron hypothesis, the mitochondrial cascade hypothesis, the vascular hypothesis, the inflammation hypothesis, the calcium dysregulation hypothesis, and others (Du et al., 2018). However, none of the hypotheses has yet been able to comprehensively explain the causes of AD.

So far, therapeutic research on AD has mainly focused on NFTs and Aβ pathology. However, drugs that specifically target these molecules have not achieved significant success. Even the FDA-approved drugs lecanemab and aducanumab, known for their ability to target Aβ, have shown limited efficacy in attenuating cognitive decline in Alzheimer’s patients (Lyu et al., 2023).

Abnormal excitability and oscillatory activity in the neuronal networks have been identified as important factors contributing to cognitive impairment in Alzheimer’s disease (Kazim et al., 2021). As a result, neuronal network dysfunction has become a significant area of interest in the diagnosis and treatment of Alzheimer’s disease (Kim et al., 2022). The entorhinal cortex (EC) is considered a vital component of hippocampal formation. It serves as a bridge between the hippocampus and the neocortex and plays a crucial role in various forms of explicit memory (Coutureau and Di Scala, 2009; Hasselmo, 2013). The EC is particularly susceptible to Alzheimer’s disease. It degenerates earlier than other brain regions and contributes significantly to the symptoms of mild cognitive impairment (MCI), such as deficits in spatial navigation (Kobro-Flatmoen et al., 2021; Igarashi, 2023). This selective vulnerability of EC in AD has greatly improved our understanding of the pathology of the disease. Consequently, the study of neuronal networks within EC may greatly expand our knowledge of the pathogenesis of AD and facilitate the discovery of new biomarkers for early diagnosis and the development of novel therapeutic targets.

This review will first provide a brief overview of the anatomical and functional organization of the EC and its dysfunction in AD. We will review studies on EC network dysfunction and conclude by discussing the modulation of neuronal activity within the EC as a potential therapeutic approach for AD.

## 2 Anatomical and functional organization of EC

### 2.1 Anatomy of EC

In addition to the hippocampus, widely acknowledged as the paramount brain region in spatial navigation and episodic memory processing, other parts of the medial temporal lobe (MTL) such as EC have attracted considerable attention, and the involvement of this area has been shown in episodic memory and spatial navigation processes within the human brain (Jacobs et al., 2010; Bellmund et al., 2020).

The EC, designated as Brodmann area 28, derives its name from a distinctive anatomical feature - the partial encirclement by the rhinal (olfactory) sulcus. This prominent feature is particularly conspicuous in nonprimate mammals, but even in primates, the anterior portion of the EC exhibits lateral demarcation by the rhinal sulcus (Witter et al., 2017; Garcia and Buffalo, 2020). It serves as a nodal point between the hippocampal formation and multimodal cortical association areas, such as the parietal, temporal, and prefrontal cortex. The EC is laterally bordered by the perirhinal cortex. Medially, the EC is adjacent to the subiculum and the hippocampus. Anteriorly, it extends toward the piriform cortex and the amygdala, while posteriorly, it transitions into the parahippocampal cortex, which is often called the postrhinal cortex in nonprimate species (Canto et al., 2008; Witter et al., 2017).

Based on their cytoarchitecture and connectivity patterns, the EC is divided into two distinct subregions in rodents, known as the lateral entorhinal cortex (LEC) and the medial entorhinal cortex (MEC) (Canto et al., 2008; Witter et al., 2017). In humans, these subregions correspond to the anterior-lateral and posterior-medial portions of the EC (Maass, 2015; Navarro Schröder et al., 2015).

Traditionally, the EC has been characterized as an intermediary structure situated between the six-layered neocortex and the three-layered archicortex (Van Groen, 2001). The EC consists of four cell-rich layers (layers II, III, V, and VI) and two relatively cell-sparse layers layers I and IV. Two types of excitatory cells reside in layer II: stellate and pyramidal cells, distinguished by their morphologies, physiological properties, projection targets, and molecular profiles. Stellate cells are likely replaced with fan cells in the LEC. Pyramidal cells dominate layers III and V (Alonso and Klink, 1993; Gerlei et al., 2021; Tukker et al., 2022). Layers II and III also house a diverse minority of approximately 10% inhibitory interneurons that release gamma-aminobutyric acid (GABA). Interneurons mainly project locally, but a small proportion also project to the hippocampus (Melzer et al., 2012; Ye et al., 2018).

**Figure 1:**
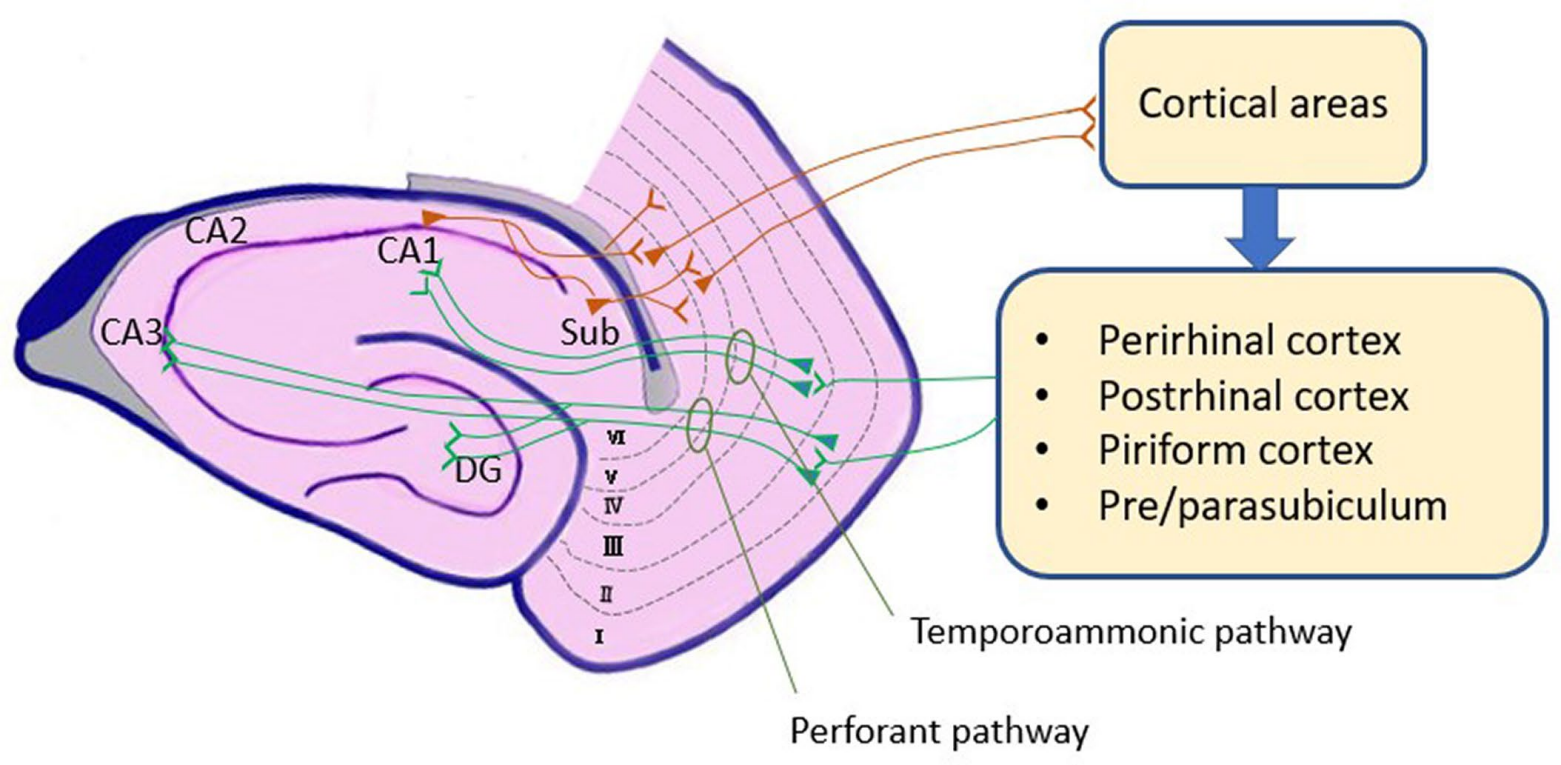
Schematic diagram of EC connections. Cortical areas connect to EC by perirhinal, postrhinal, and piriform cortex and pre/parasubiculum. Cortical inputs terminate in layers II and III of the EC. Inputs from layer II use the perforant pathway to reach the dentate gyrus or CA3, while inputs from layer III use the temporoammonic pathway to reach CA1, linking the EC with the hippocampus. Outputs from the hippocampus travel directly from CA1 or the subiculum to layers V and VI of the EC, and subsequently to cortical regions. EC, Entorhinal Cortex; DG, Dentate Gyrus; Sub, Subiculum.

The EC collects sensory information from cortical regions through connections with the perirhinal and parahippocampal cortices, and the pre-and parasubiculum. It also receives input from olfactory structures. As a result of this integration, the EC transmits these sensory inputs to different subfields within the hippocampus. Layer II neurons mostly project to the dentate gyrus and fields CA2 and CA3, while layer III neurons project to CA1 and the subiculum. In the EC, hippocampal output targets layers V and VI. In turn, they serve as the source of extensive reciprocal projections to the cortex and subcortical regions, such as the septum, striatum, amygdala, and thalamus (Maass, 2015; Witter et al., 2017). A Schematic diagram of EC connections is shown in Figure 1.

### 2.2 Functional diversity of MEC and LEC

Within the context of the influential theory postulating the existence of two separate information processing routes, it has been classically suggested that the MEC constitutes an integral component of the dorsal pathway, which specializes in processing spatial information (“where”). Conversely, the LEC is posited to be a constituent of the ventral pathway, which is responsible for processing non-spatial information (“what”) (Save and Sargolini, 2017). The MEC and LEC exhibit distinct connectivity patterns, implying a functional segregation. The main input to the MEC comes from the postrhinal cortex, whereas the LEC is mostly connected to the perirhinal cortex. Furthermore, the occipital, parietal, and cingulate areas exhibit notably greater connections with the MEC, whereas the insular, prelimbic, and infralimbic frontal regions have more prominent connections with the LEC. Both subregions establish reciprocal connections with subcortical structures, including the thalamus, amygdala, claustrum, and septum. The interconnected regions of LEC and MEC contribute to a well-structured network between EC and the hippocampus. Specifically, the dorsolateral band connects with the dorsal portion of the hippocampal formation, the intermediate band with the intermediate part, and the medial band with the ventral part. Consequently, the communication between MEC and LEC, enabled by associative connections, allows for the merging of spatial and non-spatial data at the entorhinal level (Burwell and Amaral, 1998; Pitkänen et al., 2000; Kitanishi and Matsuo, 2017).

There has been recent questioning of the idea that MEC and LEC function as completely separate entities. The hypothesis of a functional link between the two subregions is driven by findings from lesion, imaging, and electrophysiological studies. Although the general hypothesis regarding spatial vs. non-spatial distinction is not to be rejected, it is becoming increasingly clear that the concept of a strict dichotomy necessitates revision (Van Cauter et al., 2013; Save and Sargolini, 2017).

## 3 Entorhinal cortex dysfunction in AD

The EC experiences degeneration during the initial phases of AD, resulting in around 60% of layer II neurons undergoing cell death (Gómez-Isla et al., 1996; Braak and Del Tredici, 2012). In addition, EC is among the first areas where Aβ plaques accumulate (Thal et al., 2002). More importantly, the EC is particularly prone to the accumulation of NFTs as part of the natural aging process (Schöll et al., 2016; Harrison et al., 2019). The empirical study undertaken by Braak et al. demonstrates a sequential pattern of spread of NFTs in AD, beginning in the transentorhinal region and subsequently extending to other limbic regions (such as the hippocampus) and neocortical areas (Braak and Braak, 1991). The tau protein, which is the main component of NFTs, can be transferred between cells through processes such as endocytosis-exocytosis or direct membrane penetration. This phenomenon is interestingly linked to neuronal activity (Frost et al., 2009; Kfoury et al., 2012; Yamada et al., 2014; Vogels et al., 2020). Therefore, it is postulated that tau spreads throughout the brain via neuronal connections (Braak and Del Tredici, 2018). In addition, microglia have a role in promoting the spread of tau inside the brain tissue (Asai et al., 2015). Tau proteins can induce degeneration of entorhinal neurons, so breaking the connections between the hippocampus and other cortical regions, ultimately resulting in cognitive impairment observed in Alzheimer’s disease (Dickerson, 2007). While the specific reasons for the heightened vulnerability of entorhinal neurons, particularly those in layer II, to tau-related pathology and cell death in AD, are not fully known, factors such as elevated metabolic rates, neuronal plasticity, and a unique genetic profile related to tau homeostasis in this region are believed to play significant roles (Braak et al., 2006; Stranahan and Mattson, 2010; Fu et al., 2018). Given the importance of discovering the molecular mechanisms involved in the selective vulnerability of this region in AD, numerous studies have been conducted in this area. For example, the results of a study that evaluated the changes in gene expression of pyramidal neurons of different parts of the brain in aged people showed that the expression of glycogen synthase kinase 3 beta (GSK3β) and calcium/calmodulin-dependent protein kinase II delta (CaMK2D) was upregulated in the EC of the aged brains. GSK3β is an enzyme that performs a function in phosphorylating Tau and increasing CaMK2D is believed to contribute to Ca2 + −dependent toxicity which seems that both can contribute to the vulnerability of this region to AD (Liang et al., 2007). Additionally, previous research indicated that the elevated levels of reelin protein and reduced levels of calbindin, a calcium-buffering protein, in neurons of layer II of the EC make these neurons more susceptible to damage from Aβ (Thorns et al., 2001; Kobro-Flatmoen et al., 2016). In a recent study conducted using the single-nucleus RNA-sequencing (SnRNA-seq) technique in the post-mortem brains of AD, a population of EC neurons that were more vulnerable to AD was identified. Subsequent research revealed that the nuclear receptor RAR-related orphan receptor beta (RORB), which plays a crucial role in determining the identity of neuronal subtypes during development, is specifically expressed in this particular group of neurons. RORB has been discovered as a marker for neurons that are selectively vulnerable in the EC. In addition, this study also discovered a population of astrocytes with a significant downregulation of homeostatic genes in the EC region, which seems to contribute to the selective susceptibility of this region (Crist et al., 2021; Leng et al., 2021).

Given the selective vulnerability of this region to AD, numerous clinical and animal studies have reported dysfunction in the structure, histology, and neuronal networks of the EC. Notably, several studies suggest that investigating these dysfunctions could significantly aid in the early diagnosis of AD. Accordingly, Magnetic resonance imaging (MRI) studies of mild cognitive impairment (MCI) and elderly patients suggest that the degeneration and reduction of EC volume can be suitable biomarkers for predicting and early diagnosis of AD (Whitwell et al., 2007). In this regard, the identification of EC dysfunction using functional MRI (fMRI) during the performance of a virtual reality navigation test was also helpful for the detection of the early stages of AD (Kunz et al., 2015).

Table 1 summarizes clinical and preclinical studies that investigate the various facets of EC dysfunction in AD. Moreover, given that the primary focus of this review is on neural network impairments, dysfunction in the neural network of the EC region, including changes in excitability and neuronal oscillations, is discussed in greater detail in the following sections of the article.

**Table 1:** Clinical and preclinical studies on EC dysfunction in AD.

Study type	Model/group of study	Methodology	Observation(s)	Refs
Human studies				
Post-mortem	-8 control −10 MCI −11 mild or moderate AD	IHC	Decrease of the number of layer II EC neurons in MCI and AD patient Atrophy of layer II EC in MCI and AD patient	Kordower et al. (2001)
Post-mortem	−10 control −10 AD	IHC	Decrease of the number of layer II EC neurons in AD patient Layer II followed by layer IV showed the highest and earliest rates of neuronal depopulation	Gómez-Isla et al. (1996)
Post-mortem	−13 control −4 preclinical AD −8 mild symptomatic AD −4 with severe AD	IHC	Decrease of the number of layer II EC neurons in MCI and AD patient Atrophy of layer II EC in MCI and AD patient	Price et al. (2001)
Clinical study	−39 control −27 MCI −27 AD	MRI	Reduction of EC volume in MCI and AD patient EC was better than the hippocampus for distinguishing MCI from AD	Du et al. (2001)
Clinical study	−32 control −30 AD	MRI	Reduction of EC volume in AD patient	Juottonen et al. (1999)
Clinical study	−63 controls −139 MCI	MRI	Reduction of EC volumes contributes to the prediction of MCI conversion to AD	Devanand et al. (2007)
Clinical study	−59 control −65 MCI −48 AD	MRI	Reduction of EC volumes contributes to the efficient classification of MCI from healthy individuals	Pennanen et al. (2004)
Clinical study	−31 controls - 16 APOE-ε4 with AD −16 AD without APOE-ε4	MRI	Reduction of EC volume in patient with APOE-ε4 allele	Juottonen et al. (1998)
Clinical study	−33 patient with normal cognition - 17 normal cognition to MCI patient	MRI	Reduction of entorhinal and transentorhinal thickness in MCI Reduction of transentorhinal thickness predicted the MCI earlier	Kulason et al. (2020)
Clinical study	−18 control −18 probable AD	-MRI -Verbal and visuospatial episodic memory test	Reduction of EC volumes Correlation between Episodic memory impairment and reduction in the EC volumes	Di Paola et al. (2007)
Clinical study	−99 controls - 106 MCI −120 AD	MRI	Reduction of EC thickness Reduction of EC thickness could predict the decline in cognitive performance	Velayudhan et al. (2013)
Clinical study	−41 controls −45 MCI	-MRI -Virtual reality navigation task	Reduction of EC volume Impairment in the performance of entorhinal based navigational task Reduction of EC volume was correlated with impairment of navigation	Howett et al. (2019)
Clinical follow-up study	−84 controls −12 AD	fMRI	Reduction of EC CBV LEC is primarily affected in preclinical Alzheimer’s disease	Khan et al. (2014)
Clinical study	Homozygous for APOE-ε3 (“control participants”) Heterozygous for APOE-ε4/ε3 (“risk participants”) −18 male control −19 female control −18 male risk −20 female risk	-fMRI -Virtual reality navigation task	Dysfunction of the EC grid cells in APOE-ε4 carriers	Kunz et al. (2015)
Animal studies				
Experimental study	2-to 4-month-old mice −11 Wild-type −12 Tg2576	-Electrophysiology (*in vitro* slice recording) -Object placement task	Impaired performance in EC-dependent cognitive task Disruption of MEC neuronal excitability	Duffy et al. (2015)
Experimental study	3-and 6-month-old mice −17 Tg2576 −21 Wild type	Electrophysiology (*in vivo* single-unit recording in anesthetized mice)	Disruption of LEC neuronal excitability	Xu et al. (2015)
Experimental study	5-month-old mice −5 APP-KI −5 Wild type	Electrophysiology (*in vivo* recording in anesthetized mice)	Disruption of MEC gamma oscillations	Nakazono et al. (2017)
Experimental study	Young and adult mice - 38 J20 −30 Wild type	Electrophysiology (*in vivo* recording in awake mice)	Dysfunction of the MEC grid cells in adult J20 mice	Ying et al. (2022)
Experimental study	−13 APP-KI −12 Wild type	Electrophysiology (*in vivo* recording in awake mice)	Dysfunction of the MEC grid cells in APP-KI mice	Jun et al. (2020)
Experimental study	-Young and aged mice −10 Young control −9 Young EC-Tau −7 aged control −7 aged EC-Tau	-Electrophysiology (*in vivo* recording in awake mice) -IHC	Dysfunction of the MEC grid cells in aged EC-Tau mice Excitatory neuronal loss in MEC	Fu et al. (2017)
Experimental study	1, 3-, 6-, 9-, and 12-months old mice -3xTg-AD (n = 5, 4, 4, 5 and 5 respectively) -Wild type (n = 4, 4, 4, 5, 5 respectively)	IHC	Astrocytic atrophy in the EC from one-month-old age	Yeh et al. (2011)

AD, Alzheimer’s disease; EC, Entorhinal cortex; MCI, mild cognitive impairment; IHC, Immunohistochemistry; MRI, Magnetic resonance imaging, fMRI, functional Magnetic resonance imaging; LEC, Lateral Entorhinal cortex; MEC, Medial Entorhinal cortex; CBV, Cerebral Blood Volume.

### 3.1 Dysfunction of EC network excitability

#### 3.1.1 Neuronal hyperexcitability in AD

Hippocampal hyperactivity throughout memory encoding tasks in individuals with MCI and presymptomatic people that carried the E280A presenilin-1 (PS1) mutation, which is the primary driver of early-onset familial AD, has been identified by fMRI (Hector and Brouillette, 2020). Hyperexcitability of the neurons causes hyperactivity and hypersynchrony of the neural networks, which causes epileptiform and seizure activity in the early stages of AD. Seizures and epileptiform activity have been recorded in AD and MCI patients, as well as in animal models of AD (Vossel et al., 2013). Electrophysiological studies in transgenic AD animal models have reported a disturbance in neuronal network excitability, which can be related to Aβ or tau. It has been shown that in the early and presymptomatic stages of the disease, soluble Aβ damages inhibitory neurons and inhibits glutamate reuptake, which disrupts the excitatory-inhibitory balance and leads to the hyperexcitability of cortical and hippocampal neurons (Palop and Mucke, 2016; Zott et al., 2019). Hyperactivity of the cortical and hippocampal areas may play an important role in the accumulation of Aβ plaques and the spread of NFTs throughout the brain. Therefore, it can be one of the key mechanisms of disease progression and cognitive disorders (Busche and Konnerth, 2015; Targa Dias Anastacio et al., 2022). In numerous transgenic AD mouse models, neuronal hyperactivity has been identified. It was found Around 21% of the neurons in the cortex of the APP23 × PS45 animal model exhibited an elevated influx of Ca2+, primarily close to amyloid plaques (Busche et al., 2008). Moreover, hyperactivity was observed in the CA1 area of the hippocampus in young transgenic mice with AD (aged 1–2 months) at a stage when the accumulation of Aβ oligomers (Aβo) starts but before the emergence of detectable plaques (Busche et al., 2012).

These findings indicate that hyperactivity is an initial pathogenic event that depends on the accumulation of Aβo, rather than the mere existence of plaques. The plaques may serve as a storage site for harmful Aβo, thus intensifying the elevated neuronal activity that is partly responsible for the synaptic and neuronal losses found near the plaques. Alongside this hyperactivity, approximately 29% of the cortical neurons exhibited hypoactivity in mice aged 6–10 months with established plaques. It is worth mentioning that hypoactive neurons were only detected after the development of plaques. This suggests that as AD advances, the initially hyperactive neural networks eventually transition to a state of hypoactivity (Hector and Brouillette, 2020). It is noteworthy that the modulation of hyperactivity using anticonvulsant drugs such as levetiracetam improves AD-related memory disorders (Bakker et al., 2012). Although most of the neuronal hyperactivity is attributed to Aβ accumulations, the effects of tau on neuronal excitability are conflicting in different studies. However, recent studies suggest that hyperphosphorylated tau is more related to reduced neuronal excitability in the hippocampal and cortical areas (Harris et al., 2020).

#### 3.1.2 Alteration of EC neuronal excitability in AD

It is imperative to understand hippocampal and cortical excitability in AD, but understanding EC neuronal excitability also provides valuable insight into AD pathology as well. Considering this, various studies have investigated this issue. Using four-month-old Tg2576 mice carrying the Swedish mutation (APP695 with double mutations at KM670/671NL), a reduction in the firing rate was observed among LEC fan cells. The overproduction of different Aβ peptides in the MLT regions reported in this model may be involved in the hypoactivity of LEC fan cells (Marcantoni et al., 2014). In contrast, a study conducted in 3-and 6-month-old Tg2576 mice demonstrated neuronal hyperactivity in the LEC through the measurement of local field potentials and single-unit spontaneous activity in the LEC, which was correlated with amyloid precursor protein (APP) metabolites (Xu et al., 2015). Also in another study, which was conducted in Tg2576 mice, but at an earlier stage in 2 to 4-month-old mice, there was an increase in soluble Aβ expression in the LEC area. Hyperexcitability in the LEC slices was observed by increased repetitive field potentials in response to stimulus (Duffy et al., 2015). The results of these studies suggest that EC hyperexcitability may also occur before plaque deposition and thus may serve as an early indicator of AD. Other transgene models of AD have also demonstrated hyperexcitability of EC. Hyperexcitability, characterized by an elevated discharge of action potentials, increased frequency, and amplitudes of spontaneous excitatory postsynaptic potentials (sEPSPs), was also detected in the LEC of a mouse model with AD (AppNL-F/NL-F). This hyperexcitability was associated with the malfunctioning of parvalbumin (PV) interneurons and Wnt signaling (Petrache et al., 2019). Furthermore, mice expressing mutant human APP in the EC had hyperexcitable neurons. This was supported by the presence of more frequent and longer-lasting spontaneous extracellular field potentials (sEFPs) in the LEC and the occurrence of epileptiform-ictal-like discharges in the MEC. In contrast, expressing mutant human tau in the EC reduced excitability (Angulo et al., 2017). In an additional investigation, electrophysiological recordings using the whole cell patch clamp technique were conducted on stellate neurons in layer II of the MEC in three-month-old 3xTg transgenic mice, which exhibit both Aβ and tau pathologies. The results revealed that the excitability of these neurons remained unchanged in animals of this age. Additionally, no impairments in spatial memory were detected in mice at this stage. However, in mice that were 10 months old, there was a noticeable hyperexcitability in these neurons, which was accompanied by impairments in spatial memory. This suggests that spatial memory impairments may be associated with the hyperexcitability of these neurons (Chen et al., 2023). A further investigation conducted on the rTg4510 tauopathy model demonstrated a reduction in neuronal excitability in the dorsal region of the MEC, whereas the excitability of neurons in the ventral region remained unaffected (Booth et al., 2016). A reduction in grid cell firing rate was observed after the *in vivo* recording of the MEC in old EC-Tau mice with tau pathology in the MEC. Furthermore, this study found that tau pathology mostly caused neuronal death in excitatory neurons, which indicates that these neurons are more susceptible to tau than other types of neurons (Fu et al., 2017). Interestingly, the hyperexcitability of LEC pyramidal neurons was reported even in mice expressing the risk factor gene of AD (APOE4) (Nuriel et al., 2017). According to the above evidence, most evidence suggests that Aβ accumulation in the EC can cause hyperexcitability. The increase in hyperexcitability can also result in increased Aβ peptide secretion and greater plaque accumulation (Leal et al., 2017). It has been found that Aβ can facilitate tau spread in the brain, both locally and remotely (Busche and Hyman, 2020; Lee et al., 2022). Neuronal hyperactivity can also increase the release of tau from neurons which can be taken up by neighboring cells, thereby seeding even more tau to develop (Wu et al., 2016). It was found that optogenetic stimulation of the rTg4510 mice hippocampus and chemogenetic stimulation of the rTauEC mice EC increased tauopathy in mice (Schultz et al., 2018). Therefore, the increase in excitability, either directly or indirectly through the increase of Aβ facilitates the spread of tau in the hippocampal and cortical regions. As tau spreads in the brain, it can cause the disease to progress to its advanced stages and cause extensive dementia in the brain (Braak and Del Tredici, 2012). It can be therapeutically beneficial to control hyperexcitability in this area to prevent the progression of the disease.

### 3.2 Dysfunction of EC oscillatory activity

#### 3.2.1 Abnormal brain oscillatory activity in AD

Aberrant network excitability and dysfunction of inhibitory neurons can also impair the oscillatory or rhythmic activity of the brain (Palop and Mucke, 2016). Neuronal oscillations are the rhythmic fluctuation of the neuronal population’s electrical activity that can be measured by different methods such as electroencephalography (EEG), local field potentials (LFP), and magnetoencephalography (MEG). They are classified into six categories based on frequency, which includes delta (δ), theta (θ), alfa (α), beta (β), gamma (γ), and sharp-wave ripples (SWRs). Oscillatory activity in different regions of the brain enables various brain areas to connect more easily and efficiently through the synchronization of neuronal oscillations, either by coupling the phase of neuronal oscillations (phase coupling) or by coupling the amplitude of neuronal oscillations (power to power correlation) or by coupling the phase of a slower oscillation with the power of a faster one (phase-amplitude coupling), making communication faster and easier. As a result, neural oscillations are one of the basic mechanisms for processing various cognitive functions of a healthy brain, and their abnormal activity may indicate the presence of brain disorders such as AD (Ward, 2003; Buzsáki and Watson, 2012).

Dysfunction in neural networks oscillatory activities plays a critical role in the pathology and clinical manifestations of AD (Aron and Yankner, 2016). It occurs in the early phase of a pathogenic cascade that leads to exacerbation of AD progression and finally disruption of neural circuits that underlie higher cognitive functions in affected individuals (Canter et al., 2016). Therefore, several EEG investigations that encompassed individuals with both MCI and AD have consistently observed alterations in brain oscillations compared with a group of healthy individuals. These alterations include a reduction in alpha and beta activity, along with an increase in delta and theta activity. In addition, it has been observed that decreased complexity and coherence in EEG recordings could be used as biomarkers for diagnosing AD (Le Roc’h, 1994; Stam et al., 2003). Decreased EEG synchronization and loss of oscillatory activity, particularly of gamma-frequency oscillations, have been observed in MCI and AD patients (Koenig et al., 2005).

Due to the need for invasive methods for studying the neuronal oscillations of subcortical areas, mouse rodent animal models are used. In this regard, the recording of electrophysiological signals from hippocampal areas of rodent AD models shows that gamma and theta oscillations and the phase-amplitude coupling between them (theta–gamma coupling) are more disturbed during AD (Mehak et al., 2022). Theta, gamma, and SWRs are oscillatory activities that are involved in various memory processes, including encoding, consolidation, and retrieval (Düzel et al., 2010; Joo and Frank, 2018). A decrease in the power of the low gamma frequency band has been reported in the hippocampus of many AD animal models. Studies suggest that soluble Aβ peptides cause damage to PV neurons, Considering the important role of PV neurons in generating gamma oscillations, they disrupt the generation of these oscillations (Verret et al., 2012; Martinez-Losa et al., 2018). It is important to note that, impairment of the hippocampal gamma oscillatory activity band has been reported even before the accumulation of Aβ plaques (Iaccarino et al., 2016). For this reason, gamma oscillations have attracted a lot of attention in AD research. Therefore, restoring gamma oscillations is proposed as one of the solutions for the treatment of AD (Adaikkan and Tsai, 2020). In this regard, preclinical studies have shown that the entrainment of 40 Hz gamma oscillations by using different techniques, including optogenetic stimulation, sensory visual and auditory stimulation, and transcranial-focused ultrasound has shown effective results in improving AD-like pathologies (Adaikkan et al., 2019; Martorell et al., 2019; Park et al., 2021).

Overall, it can be deduced that the impairment of neuronal oscillations is an important component for examining the normal activity of the neural network in AD and is considered a biomarker for AD early diagnosis and a potential therapeutic target.

#### 3.2.2 Alteration of EC oscillatory activity in AD

The proper functioning of the EC during spatial navigation relies on its oscillatory dynamics. The main neuronal oscillations in the EC are gamma and theta, which play critical roles in transferring spatial information to the hippocampus (Quilichini et al., 2010). Theta-gamma coupling is an essential phenomenon for accurate coordination of the hippocampus and EC during the process of encoding and retrieval of memory. It involves the emergence of gamma oscillations at specific phases of theta oscillations (Colgin, 2015). Considering the importance of EC oscillatory activity in memory, disturbance in EC oscillatory activity can result in the dysfunction of various memory processes. This has motivated various studies to investigate neuronal oscillations in EC using animal models of AD. One of the first studies conducted on this issue was conducted using amyloid precursor protein-knock-in mice (APP-KI). LFP recordings in the MEC of APP-KI mice demonstrated impaired theta-fast gamma coupling and spike phase locking of pyramidal neurons. However, the power of gamma oscillations and theta oscillations was not affected. The disruption of gamma temporal organization in the MEC is evident from these findings (Nakazono et al., 2017). In a subsequent study, MEC grid cells were found to have poor spatial tuning in aged APP-KI mice with memory disorders. Furthermore, fast gamma oscillations, which are crucial to transmitting information to the hippocampus, were also disrupted. Consequently, there was a noticeable reduction in gamma coherence between the MEC and the hippocampus. When MEC activity was recorded in young APP KI mice who did not yet have significant cognitive disorders, grid cells showed abnormal spatial tuning. In contrast, hippocampal place cells did not show abnormal tuning. This result indicated that MEC dysfunction occurs earlier in this disease than in other areas, and most spatial memory disorders originate here (Jun et al., 2020).

**Figure 2:**
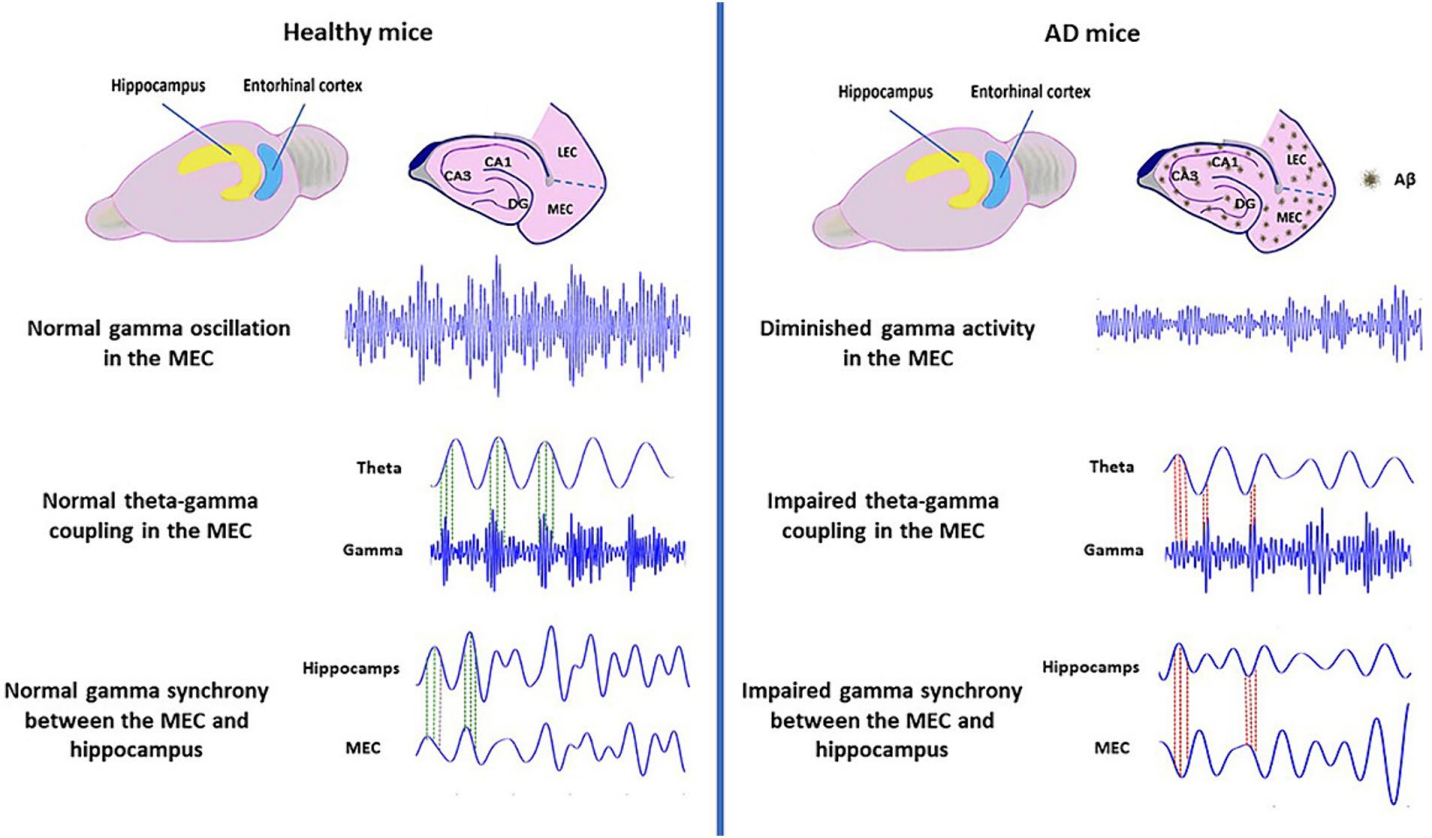
Alterations of the MEC oscillatory activity in Aβ-based models of AD. Impairment of gamma oscillations, theta-gamma coupling, and MEC-hippocampal gamma synchrony based on mouse models of AD. AD, Alzheimer’s disease; MEC, Medial Entorhinal cortex; DG, Dentate Gyrus.

Researchers conducted a recent study on J20 male mice and found a dysfunctional pattern in the grid cells. The pattern was characterized by reduced spatial stability and lack of synchronization with head direction cells and associated with poor path integration (Ying et al., 2022). Video EEG recordings of MEC in 6-month-old APP KI mice showed impaired low gamma power. These mice were also recorded during SWRs in the MEC and CA1, revealing disrupted synchronization in the early stages of the disease. Coordination between the MEC and CA1 may support hippocampal long-duration SWRs. Disruption of this synchronization during SWRs could contribute to later memory consolidation dysfunction in AD, as these long-duration SWRs are essential for memory consolidation (Funane et al., 2022). Consequently, the main alterations observed in Aβ-based models include diminished power and temporal coherence of gamma oscillations, as well as disrupted connectivity between the MEC and hippocampus, which is primarily attributed to disturbances in gamma oscillations (Figure 2). Researchers have also investigated the oscillatory activity of tau-based models in EC-tau mice. This model showed an increase in theta activity in this region. This may be due to the loss of excitatory neurons, which is consistent with a higher level of theta rhythm in patients with mild cognitive impairment in the early stages of AD (Fu et al., 2017). Further research on the rTg4510 tauopathy transgenic mice showed a disruption in gamma oscillations, particularly in the dorsal region of the MEC, but no significant change in the oscillatory activity of the ventral part (Booth et al., 2016).

LEC is regarded as a critical node of AD (Mandino et al., 2022). However, little research has been conducted on the alterations in oscillatory activity within this area. A recent study discovered that injecting Aβ i.c.v. resulted in the aggregation of plaques in the LEC and disrupted synchronization between the LEC, olfactory bulb, and hippocampal area. This synchronization impairment has been linked to a decline in recognition memory during testing for novel object recognition (Salimi et al., 2022).

## 4 Modulation of EC neuronal activity as a therapeutic target for AD

The entorhinal-hippocampal system, regarded as the brain’s major memory hub, investigating whether external modulation of this circuit can be effective in improving memory has become one of the challenges of neuroscientists. Human studies have revealed that deep brain and theta-burst stimulation in the entorhinal region during memory tasks can improve memory performance (Titiz et al., 2017), while memory impairment was also reported in some studies (Jacobs et al., 2016). Conflicting outcomes may arise from differences in stimulation sites within the entorhinal region (Mankin and Fried, 2020). Moreover, animal studies also implicated that electrical or optogenetic stimulation of the EC can enhance neurogenesis and spatial memory (Stone et al., 2011; Chavoshinezhad et al., 2021). Overall, the collective evidence underscores the EC’s neuromodulation as a promising avenue for manipulating memory processes (Mankin and Fried, 2020).

According to the evidence discussed in the previous sections, pathological outcomes of AD can lead to impairment of both network excitability and oscillatory dynamics of the EC. Therefore, the modulation of neuronal activity in the EC region has been proposed as a therapeutic goal for AD. Reduction of EC neuronal hyperactivity through chemogenic stimulation has been found to prevent the spread of tau to the hippocampal regions in transgenic mice expressing both Aβ and tau in the EC (Rodriguez et al., 2020). This suggests that regulating EC hyperactivity could be a useful strategy in preventing the widespread distribution of tau and delaying the onset of the degenerative phase of AD. Various neuromodulation techniques, including deep brain stimulation (DBS), have also been shown to be effective in AD treatment by restoring the disrupted oscillatory activity of the EC area. Research employing AD transgenic mice revealed that 25 days of 130 Hz DBS in the EC enhanced hippocampal neurogenesis, decreased tau and Aβ pathology, and improved memory problems in 3xTg mice (Mann et al., 2018). Additionally, 130 Hz DBS in the EC for 6 weeks was found to be effective in treating memory disorders and Aβ pathology in aged and young TgCRND8 mice (Xia et al., 2017). According to a recent study, 21 days of 10 Hz DBS not only improved pathological hallmarks of AD but also could restore impaired hippocampal theta and gamma power and theta-high gamma coupling, suggesting the crucial role of the EC neuronal activity in regulating hippocampal neural networks (Luo et al., 2023). These results suggest that modulating EC neuronal activity may be a promising therapeutic target for AD. Despite this, using neuromodulation in this area as a therapeutic method in humans presents numerous challenges. It is imperative to investigate the therapeutic effects of these therapies on tauopathy, given the region’s high susceptibility to it. Up to now, preclinical studies have primarily been conducted on transgenic rodent models. However, due to differences in the expression of tau isoforms between humans and rodents, the tauopathy observed in rodents does not fully resemble that in humans (Hernández et al., 2020; Sahara and Yanai, 2023). Thus, additional research with more suitable tau pathology models is required. Furthermore, since the EC region suffers significant damage during AD, focusing on this area as a therapeutic target faces considerable temporal and anatomical limitations and requires precise preclinical studies. It is important to note that the progress in non-invasive techniques for deep brain stimulation, such as targeted ultrasound, temporal interference, near-infrared optogenetics, and nanomaterial-enabled magnetic stimulation (Liu et al., 2022), holds great potential for stimulating this specific region.

## 5 Conclusion

EC is one of the most critical areas involved in episodic memory. Neuroimaging studies in individuals with Alzheimer’s and MCI indicate that this region is affected by structural and functional disturbances in the early stages of the disease. In addition, electrophysiological studies in animal models have abundantly reported dysfunction in this area, especially its grid cells. Given the initiation of tauopathy from this region, it is considered a strategic area in the treatment and prevention of this disease.

Exploring the disruption of brain network activity is a cutting-edge approach in Alzheimer’s research. Numerous studies have demonstrated a connection between neuronal hyperactivity and abnormal oscillatory rhythmic activity in the hippocampus and cognitive dysfunction in AD. Many animal studies have reported neuronal hyperexcitability in the EC, especially in the LEC, in the early stages of AD. In most cases, hyperexcitability has been associated with the accumulation of Aβ in this area. Studies suggest that hyperexcitability in this region leads to the spread of tau to other brain areas, consequently contributing to disease progression. Therefore, modulating the hyperexcitability of this region may be a therapeutic target for AD. Further research is needed to elucidate the mechanisms involved in the hyperexcitability of this area. For instance, studying inhibitory neurons, ion channels, and neurotransmitters in this region can be informative. Fewer studies have addressed changes in excitability in the MEC; therefore, there is a need for further research in this area.

Aberrant oscillatory activity is a critical aspect of AD pathology. The disruption of neural oscillations, particularly gamma and theta frequencies, has a significant impact on cognitive functions. Studying the neuronal oscillations in the MEC region of AD animal models, through LFP recording, indicates a disruption in the dynamics of gamma oscillations in that specific area. Considering the importance of gamma activity in synchronizing different components of hippocampal formations, disruption in gamma oscillations can lead to disturbances in the synchrony of the hippocampal-entorhinal circuit. Given the critical role of hippocampal-entorhinal connectivity in memory, disruption in this circuitry may be associated with AD cognitive impairments. Further research is needed to determine whether restoring gamma oscillations in this region yields therapeutic effects. The alterations in LEC oscillations during AD lack comprehensive investigation, emphasizing the necessity for further research in this area.

Ultimately, given the promising outcomes observed in preclinical AD studies using deep brain stimulation for neuronal modulation in this area, exploring network disruptions in this region can significantly enhance the refinement of neuromodulation therapies.

## Author contributions

FK: Conceptualization, Investigation, Methodology, Visualization, Writing – original draft. AA: Investigation, Validation, Writing – review & editing. M-RA-D: Writing – review & editing. M-SS: Writing – review & editing. LD: Funding acquisition, Supervision, Validation, Writing – review & editing.

## References

[ref1] Adaikkan C. Middleton S. J. Marco A. Pao P. C. Mathys H. Kim D. N. W.. (2019). Gamma entrainment binds higher-order brain regions and offers neuroprotection. Neuron 102, 929–943.e8. doi: 10.1016/j.neuron.2019.04.011, PMID: 31076275 PMC6697125

[ref2] Adaikkan C. Tsai L.-H. (2020). Gamma entrainment: impact on Neurocircuits, glia, and therapeutic opportunities. Trends Neurosci. 43, 24–41. doi: 10.1016/j.tins.2019.11.001, PMID: 31836315

[ref3] Alonso A. Klink R. (1993). Differential electroresponsiveness of stellate and pyramidal-like cells of medial entorhinal cortex layer II. J. Neurophysiol. 70, 128–143. doi: 10.1152/jn.1993.70.1.128, PMID: 8395571

[ref4] Alzheimer’s Disease (2021). Alzheimer’s disease facts and figures’. Alzheimers Dement. 17, 327–406. doi: 10.1002/alz.12328, PMID: 33756057

[ref5] Angulo S. L. Orman R. Neymotin S. A. Liu L. Buitrago L. Cepeda-Prado E.. (2017). Tau and amyloid-related pathologies in the entorhinal cortex have divergent effects in the hippocampal circuit. Neurobiol. Dis. 108, 261–276. doi: 10.1016/j.nbd.2017.08.015, PMID: 28860088

[ref6] Aron L. Yankner B. A. (2016). Neurodegenerative disorders: neural synchronization in Alzheimer’s disease. Nature 540, 207–208. doi: 10.1038/540207a 27929001

[ref7] Asai H. Ikezu S. Tsunoda S. Medalla M. Luebke J. Haydar T.. (2015). Depletion of microglia and inhibition of exosome synthesis halt tau propagation. Nat. Neurosci. 18, 1584–1593. doi: 10.1038/nn.4132, PMID: 26436904 PMC4694577

[ref8] Bakker A. Krauss G. L. Albert M. S. Speck C. L. Jones L. R. Stark C. E.. (2012). Reduction of hippocampal hyperactivity improves cognition in amnestic mild cognitive impairment. Neuron 74, 467–474. doi: 10.1016/j.neuron.2012.03.023, PMID: 22578498 PMC3351697

[ref9] Bellmund J. L. S. Polti I. Doeller C. F. (2020). Sequence memory in the hippocampal-entorhinal region. J. Cogn. Neurosci. 32, 2056–2070. doi: 10.1162/jocn_a_01592, PMID: 32530378

[ref10] Booth C. A. Ridler T. Murray T. K. Ward M. A. de Groot E. Goodfellow M.. (2016). Electrical and network neuronal properties are preferentially disrupted in dorsal, but not ventral, medial entorhinal cortex in a mouse model of Tauopathy. J. Neurosci. Off. J. Soc. Neurosci. 36, 312–324. doi: 10.1523/JNEUROSCI.2845-14.2016, PMID: 26758825 PMC4710763

[ref11] Braak H. Del Tredici K. (2012). Alzheimer’s disease: pathogenesis and prevention. Alzheimers Dement. 8, 227–233. doi: 10.1016/j.jalz.2012.01.011 22465174

[ref12] Braak H. Del Tredici K. (2018). Spreading of tau pathology in sporadic Alzheimer’s disease along Cortico-cortical top-down connections. Cerebral Cortex 28, 3372–3384. doi: 10.1093/cercor/bhy152, PMID: 29982389 PMC6095209

[ref13] Braak H. Rüb U. Schultz C. Tredici K. D. (2006). Vulnerability of cortical neurons to Alzheimer’s and Parkinson’s diseases. Journal of Alzheimer’s disease: JAD 9, 35–44. doi: 10.3233/jad-2006-9s305 16914843

[ref500] Braak H. Braak E. (1991). ‘Neuropathological stageing of Alzheimer-related changes’. Acta Neuropathologica, 82:239259. doi: 10.1007/BF00308809, PMID: 1759558

[ref14] Burwell R. D. Amaral D. G. (1998). Perirhinal and postrhinal cortices of the rat: interconnectivity and connections with the entorhinal cortex. J. Comp. Neurol. 391, 293–321. doi: 10.1002/(sici)1096-9861(19980216)391:3<293::aid-cne2>3.0.co;2-x, PMID: 9492202

[ref15] Busche M. A. Chen X. Henning H. A. Reichwald J. Staufenbiel M. Sakmann B.. (2012). Critical role of soluble amyloid-β for early hippocampal hyperactivity in a mouse model of Alzheimer’s disease. Proc. Natl. Acad. Sci. U. S. A. 109, 8740–8745. doi: 10.1073/pnas.1206171109, PMID: 22592800 PMC3365221

[ref16] Busche M. A. Eichhoff G. Adelsberger H. Abramowski D. Wiederhold K. H. Haass C.. (2008). Clusters of hyperactive neurons near amyloid plaques in a mouse model of Alzheimer’s disease. Science 321, 1686–1689. doi: 10.1126/science.1162844 18802001

[ref17] Busche M. A. Hyman B. T. (2020). Synergy between amyloid-β and tau in Alzheimer’s disease. Nat. Neurosci. 23, 1183–1193. doi: 10.1038/s41593-020-0687-6 32778792 PMC11831977

[ref18] Busche M. A. Konnerth A. (2015). Neuronal hyperactivity--a key defect in Alzheimer’s disease? Bio Essays 37, 624–632. doi: 10.1002/bies.201500004 25773221

[ref19] Buzsáki G. Watson B. O. (2012). Brain rhythms and neural syntax: implications for efficient coding of cognitive content and neuropsychiatric disease. Dialogues Clin. Neurosci. 14, 345–367. doi: 10.31887/DCNS.2012.14.4/gbuzsaki, PMID: 23393413 PMC3553572

[ref20] Canter R. G. Penney J. Tsai L.-H. (2016). The road to restoring neural circuits for the treatment of Alzheimer’s disease. Nature 539, 187–196. doi: 10.1038/nature20412, PMID: 27830780

[ref21] Canto C. B. Wouterlood F. G. Witter M. P. (2008). What does the anatomical organization of the entorhinal cortex tell us? Neural Plast. 2008:381243. doi: 10.1155/2008/381243, PMID: 18769556 PMC2526269

[ref22] Chavoshinezhad S. Zibaii M. I. Seyed Nazari M. H. Ronaghi A. Asgari Taei A. Ghorbani A.. (2021). Optogenetic stimulation of entorhinal cortex reveals the implication of insulin signaling in adult rat’s hippocampal neurogenesis. Prog. Neuro-Psychopharmacol. Biol. Psychiatry 111:110344. doi: 10.1016/j.pnpbp.2021.110344, PMID: 33964323

[ref23] Chen L. Christenson Wick Z. Vetere L. M. Vaughan N. Jurkowski A. Galas A.. (2023). Progressive excitability changes in the medial entorhinal cortex in the 3xTg mouse model of Alzheimer’s disease pathology. J. Neurosci. 43, 7441–7454. doi: 10.1523/JNEUROSCI.1204-23.2023, PMID: 37714705 PMC10621765

[ref24] Colgin L. L. (2015). Theta-gamma coupling in the entorhinal-hippocampal system. Curr. Opin. Neurobiol. 31, 45–50. doi: 10.1016/j.conb.2014.08.001, PMID: 25168855 PMC4340819

[ref502] Coutureau E. Di Scala G. (2009). Entorhinal cortex and cognition. Progress in Neuro-Psychopharmacology and Biological Psychiatry, 33:753761. doi: 10.1016/j.pnpbp.2009.03.038, PMID: 19376185

[ref25] Crist A. M. Hinkle K. M. Wang X. Moloney C. M. Matchett B. J. Labuzan S. A.. (2021). Transcriptomic analysis to identify genes associated with selective hippocampal vulnerability in Alzheimer’s disease. Nat. Commun. 12:2311. doi: 10.1038/s41467-021-22399-3, PMID: 33875655 PMC8055900

[ref26] Devanand D. P. Pradhaban G. Liu X. Khandji A. de Santi S. Segal S.. (2007). Hippocampal and entorhinal atrophy in mild cognitive impairment: prediction of Alzheimer disease. Neurology 68, 828–836. doi: 10.1212/01.wnl.0000256697.20968.d7 17353470

[ref27] Di Paola M. Macaluso E. Carlesimo G. A. Tomaiuolo F. Worsley K. J. Fadda L.. (2007). Episodic memory impairment in patients with Alzheimer’s disease is correlated with entorhinal cortex atrophy. A voxel-based morphometry study. J. Neurol. 254, 774–781. doi: 10.1007/s00415-006-0435-1, PMID: 17404777

[ref28] Dickerson B. C. (2007). The entorhinal cortex: an anatomical mediator of genetic vulnerability to Alzheimer’s disease? Lancet Neurol. 6, 471–473. doi: 10.1016/S1474-4422(07)70112-6, PMID: 17509474

[ref29] Du A. T. Schuff N. Amend D. Laakso M. P. Hsu Y. Y. Jagust W. J.. (2001). Magnetic resonance imaging of the entorhinal cortex and hippocampus in mild cognitive impairment and Alzheimer’s disease. J. Neurol. Neurosurg. Psychiatry 71, 441–447. doi: 10.1136/jnnp.71.4.441, PMID: 11561025 PMC1763497

[ref30] Du X. Wang X. Geng M. (2018). Alzheimer’s disease hypothesis and related therapies. Transl. Neurodegener. 7:2. doi: 10.1186/s40035-018-0107-y 29423193 PMC5789526

[ref31] Duffy A. M. Morales-Corraliza J. Bermudez-Hernandez K. M. Schaner M. J. Magagna-Poveda A. Mathews P. M.. (2015). Entorhinal cortical defects in Tg2576 mice are present as early as 2-4 months of age. Neurobiol. Aging 36, 134–148. doi: 10.1016/j.neurobiolaging.2014.07.001, PMID: 25109765 PMC4268389

[ref32] Düzel E. Penny W. D. Burgess N. (2010). Brain oscillations and memory. Curr. Opin. Neurobiol. 20, 143–149. doi: 10.1016/j.conb.2010.01.004 20181475

[ref33] Frost B. Jacks R. L. Diamond M. I. (2009). Propagation of tau Misfolding from the outside to the inside of a cell *. J. Biol. Chem. 284, 12845–12852. doi: 10.1074/jbc.M808759200, PMID: 19282288 PMC2676015

[ref34] Fu H. Hardy J. Duff K. E. (2018). Selective vulnerability in neurodegenerative diseases. Nat. Neurosci. 21, 1350–1358. doi: 10.1038/s41593-018-0221-2, PMID: 30250262 PMC6360529

[ref35] Fu H. Rodriguez G. A. Herman M. Emrani S. Nahmani E. Barrett G.. (2017). Tau pathology induces excitatory neuron loss, grid cell dysfunction, and spatial memory deficits reminiscent of early Alzheimer’s disease. Neuron 93, 533–541.e5. doi: 10.1016/j.neuron.2016.12.023, PMID: 28111080 PMC5363269

[ref36] Funane T. Jun H. Sutoko S. Saido T. C. Kandori A. Igarashi K. M. (2022). Impaired sharp-wave ripple coordination between the medial entorhinal cortex and hippocampal CA1 of knock-in model of Alzheimer’s disease. Front. Syst. Neurosci. 16:955178. doi: 10.3389/fnsys.2022.955178, PMID: 36090186 PMC9452631

[ref37] Garcia A. D. Buffalo E. A. (2020). Anatomy and function of the primate entorhinal cortex. Ann. Rev. Vision Sci. 6, 411–432. doi: 10.1146/annurev-vision-030320-041115 PMC12889097 32580662

[ref38] Gerlei K. Z. Brown C. M. Sürmeli G. Nolan M. F. (2021). Deep entorhinal cortex: from circuit organization to spatial cognition and memory. Trends Neurosci. 44, 876–887. doi: 10.1016/j.tins.2021.08.003, PMID: 34593254

[ref39] Gómez-Isla T. Price J. L. McKeel Jr D. W. Morris J. C. Growdon J. H. Hyman B. T. (1996). Profound loss of layer II entorhinal cortex neurons occurs in very mild Alzheimer’s disease. J. Neurosci. 16, 4491–4500. doi: 10.1523/JNEUROSCI.16-14-04491.1996, PMID: 8699259 PMC6578866

[ref40] Harris S. S. Wolf F. de Strooper B. Busche M. A. (2020). Tipping the scales: peptide-dependent dysregulation of neural circuit dynamics in Alzheimer’s disease. Neuron 107, 417–435. doi: 10.1016/j.neuron.2020.06.005, PMID: 32579881

[ref501] Harrison T. M. Joie R. L. Baker S. L. Swinnerton K. Fenton L.. (2019). Longitudinal tau accumulation and atrophy in aging and alzheimer disease’. Annals of Neurology, 85:229240. doi: 10.1002/ana.25406, PMID: 30597624 PMC6579738

[ref41] Hasselmo E. M. (2013). How we remember brain mechanisms of episodic memory. Cambridge, MA: The MIT Press.

[ref42] Hector A. Brouillette J. (2020). Hyperactivity induced by soluble amyloid-β oligomers in the early stages of Alzheimer’s disease. Front. Mol. Neurosci. 13:600084. doi: 10.3389/fnmol.2020.600084, PMID: 33488358 PMC7817907

[ref43] Hernández F. Merchán-Rubira J. Vallés-Saiz L. Rodríguez-Matellán A. Avila J. (2020). Differences between human and murine tau at the N-terminal end. Front. Aging Neurosci. 12:11. doi: 10.3389/fnagi.2020.00011, PMID: 32063841 PMC6999090

[ref44] Howett D. Castegnaro A. Krzywicka K. Hagman J. Marchment D. Henson R.. (2019). ‘Differentiation of mild cognitive impairment using an entorhinal cortex-based test of virtual reality navigation. brain: a. J. Neurol. 142, 1751–1766. doi: 10.1093/brain/awz116, PMID: 31121601 PMC6536917

[ref45] Iaccarino H. F. Singer A. C. Martorell A. J. Rudenko A. Gao F. Gillingham T. Z.. (2016). Gamma frequency entrainment attenuates amyloid load and modifies microglia. Nature 540, 230–235. doi: 10.1038/nature20587, PMID: 27929004 PMC5656389

[ref46] Igarashi K. M. (2023). Entorhinal cortex dysfunction in Alzheimer’s disease. Trends Neurosci. 46, 124–136. doi: 10.1016/j.tins.2022.11.006, PMID: 36513524 PMC9877178

[ref47] Jacobs J. Kahana M. J. Ekstrom A. D. Mollison M. V. Fried I. (2010). A sense of direction in human entorhinal cortex. Proc. Natl. Acad. Sci. USA 107, 6487–6492. doi: 10.1073/pnas.0911213107, PMID: 20308554 PMC2851993

[ref48] Jacobs J. Miller J. Lee S. A. Coffey T. Watrous A. J. Sperling M. R.. (2016). Direct electrical stimulation of the human entorhinal region and Hippocampus impairs memory. Neuron 92, 983–990. doi: 10.1016/j.neuron.2016.10.062, PMID: 27930911

[ref49] Joo H. R. Frank L. M. (2018). The hippocampal sharp wave-ripple in memory retrieval for immediate use and consolidation. Nat. Rev. Neurosci. 19, 744–757. doi: 10.1038/s41583-018-0077-1, PMID: 30356103 PMC6794196

[ref50] Jun H. Bramian A. Soma S. Saito T. Saido T. C. Igarashi K. M. (2020). Disrupted place cell remapping and impaired grid cells in a Knockin model of Alzheimer’s disease. Neuron 107, 1095–1112.e6. doi: 10.1016/j.neuron.2020.06.023, PMID: 32697942 PMC7529950

[ref51] Juottonen K. Laakso M. P. Partanen K. Soininen H. (1999). Comparative MR analysis of the entorhinal cortex and hippocampus in diagnosing Alzheimer disease. AJNR Am. J. Neuroradiol. 20, 139–144, PMID: 9974069

[ref52] Juottonen K. Lehtovirta M. Helisalmi S. Sr P. J. R. Soininen H. (1998). Major decrease in the volume of the entorhinal cortex in patients with Alzheimer’s disease carrying the apolipoprotein E ε4 allele. J. Neurol. Neurosurg. Psychiatry 65, 322–327. doi: 10.1136/jnnp.65.3.322, PMID: 9728943 PMC2170244

[ref53] Kazim S. F. Seo J. H. Bianchi R. Larson C. S. Sharma A. Wong R. K. S.. (2021). Neuronal network excitability in Alzheimer’s disease: the puzzle of similar versus divergent roles of amyloid β and tau. eNeuro 8:ENEURO.0418. doi: 10.1523/ENEURO.0418-20.2020, PMID: 33741601 PMC8174042

[ref54] Kfoury N. Holmes B. B. Jiang H. Holtzman D. M. Diamond M. I. (2012). Trans-cellular propagation of tau aggregation by Fibrillar species *. J. Biol. Chem. 287, 19440–19451. doi: 10.1074/jbc.M112.346072, PMID: 22461630 PMC3365982

[ref55] Khan U. A. Liu L. Provenzano F. A. Berman D. E. Profaci C. P. Sloan R.. (2014). Molecular drivers and cortical spread of lateral entorhinal cortex dysfunction in preclinical Alzheimer’s disease. Nat. Neurosci. 17, 304–311. doi: 10.1038/nn.3606, PMID: 24362760 PMC4044925

[ref56] Kim S. Nam Y. Kim H. Jung H. Jeon S. G. Hong S. B.. (2022). Alteration of neural pathways and its implications in Alzheimer’s disease. Biomedicines 10:845. doi: 10.3390/biomedicines10040845, PMID: 35453595 PMC9025507

[ref57] Kitanishi T. Matsuo N. (2017). Organization of the Claustrum-to-Entorhinal Cortical Connection in mice. J. Neurosci. Off. J. Soc. Neurosci. 37, 269–280. doi: 10.1523/JNEUROSCI.1360-16.2016, PMID: 28077707 PMC6596572

[ref58] Knopman D. S. Amieva H. Petersen R. C. Chételat G. Holtzman D. M. Hyman B. T.. (2021). Alzheimer disease. Nat. Rev. Dis. Primers 7:33. doi: 10.1038/s41572-021-00269-y, PMID: 33986301 PMC8574196

[ref59] Kobro-Flatmoen A. Lagartos-Donate M. J. Aman Y. Edison P. Witter M. P. Fang E. F. (2021). Re-emphasizing early Alzheimer’s disease pathology starting in select entorhinal neurons, with a special focus on mitophagy. Ageing Res. Rev. 67:101307. doi: 10.1016/j.arr.2021.101307, PMID: 33621703

[ref60] Kobro-Flatmoen A. Nagelhus A. Witter M. P. (2016). Reelin-immunoreactive neurons in entorhinal cortex layer II selectively express intracellular amyloid in early Alzheimer’s disease. Neurobiol. Dis. 93, 172–183. doi: 10.1016/j.nbd.2016.05.012, PMID: 27195475

[ref61] Koenig T. Prichep L. Dierks T. Hubl D. Wahlund L. O. John E. R.. (2005). Decreased EEG synchronization in Alzheimer’s disease and mild cognitive impairment. Neurobiol. Aging 26, 165–171. doi: 10.1016/j.neurobiolaging.2004.03.008 15582746

[ref62] Kordower J. H. Chu Y. Stebbins G. T. DeKosky S. T. Cochran E. J. Bennett D.. (2001). Loss and atrophy of layer II entorhinal cortex neurons in elderly people with mild cognitive impairment. Ann. Neurol. 49, 202–213. doi: 10.1002/1531-8249(20010201)49:2<202::AID-ANA40>3.0.CO;2-3, PMID: 11220740

[ref63] Kulason S. Xu E. Tward D. J. Bakker A. Albert M. Younes L.. (2020). Entorhinal and Transentorhinal atrophy in preclinical Alzheimer’s disease. Front. Neurosci. 14:804. doi: 10.3389/fnins.2020.00804, PMID: 32973425 PMC7472871

[ref64] Kunz L. Schröder T. N. Lee H. Montag C. Lachmann B. Sariyska R.. (2015). Reduced grid-cell-like representations in adults at genetic risk for Alzheimer’s disease. Science 350, 430–433. doi: 10.1126/science.aac8128 26494756

[ref65] Le Roc’h K. (1994). EEG coherence in Alzheimer disease, by Besthorn et al. Electroencephalogr. Clin. Neurophysiol. 91, 232–233, 7522153 10.1016/0013-4694(94)90074-4

[ref66] Leal S. L. Landau S. M. Bell R. K. Jagust W. J. (2017). Hippocampal activation is associated with longitudinal amyloid accumulation and cognitive decline. eLife 6:e22978. doi: 10.7554/eLife.22978 28177283 PMC5325620

[ref67] Lee W. J. Brown J. A. Kim H. R. la Joie R. Cho H. Lyoo C. H.. (2022). Regional Aβ-tau interactions promote onset and acceleration of Alzheimer’s disease tau spreading. Neuron 110, 1932–1943.e5. doi: 10.1016/j.neuron.2022.03.034, PMID: 35443153 PMC9233123

[ref68] Leng K. Li E. Eser R. Piergies A. Sit R. Tan M.. (2021). Molecular characterization of selectively vulnerable neurons in Alzheimer’s disease. Nat. Neurosci. 24, 276–287. doi: 10.1038/s41593-020-00764-7, PMID: 33432193 PMC7854528

[ref69] Liang W. S. Dunckley T. Beach T. G. Grover A. Mastroeni D. Walker D. G.. (2007). Gene expression profiles in anatomically and functionally distinct regions of the normal aged human brain. Physiol. Genomics 28, 311–322. doi: 10.1152/physiolgenomics.00208.2006, PMID: 17077275 PMC2259385

[ref70] Liu X. Qiu F. Hou L. Wang X. (2022). Review of noninvasive or minimally invasive deep brain stimulation. Front. Behav. Neurosci. 15:17. doi: 10.3389/fnbeh.2021.820017, PMID: 35145384 PMC8823253

[ref71] Luo Y. Sun Y. Wen H. Wang X. Zheng X. Ge H.. (2023). Deep brain stimulation of the entorhinal cortex modulates CA1 theta-gamma oscillations in mouse models of preclinical Alzheimer’s disease. Biocybern. Biomed. Eng. 43, 246–260. doi: 10.1016/j.bbe.2022.12.010

[ref72] Lyu D. Lyu X. Huang L. Fang B. (2023). Effects of three kinds of anti-amyloid-β drugs on clinical, biomarker, neuroimaging outcomes and safety indexes: a systematic review and meta-analysis of phase II/III clinical trials in Alzheimer’s disease. Ageing Res. Rev. 88:101959. doi: 10.1016/j.arr.2023.101959, PMID: 37217078

[ref73] Maass A. (2015). Functional subregions of the human entorhinal cortex. eLife 4:e06426. doi: 10.7554/eLife.06426 26052749 PMC4458841

[ref74] Mandino F. Yeow L. Y. Bi R. Sejin L. Bae H. G. Baek S. H.. (2022). The lateral entorhinal cortex is a hub for local and global dysfunction in early Alzheimer’s disease states. J. Cereb. Blood Flow Metab. 42, 1616–1631. doi: 10.1177/0271678X221082016, PMID: 35466772 PMC9441719

[ref75] Mankin E. A. Fried I. (2020). Modulation of human memory by deep brain stimulation of the entorhinal-hippocampal circuitry. Neuron 106, 218–235. doi: 10.1016/j.neuron.2020.02.024, PMID: 32325058 PMC7347298

[ref76] Mann A. Gondard E. Tampellini D. Milsted J. A. T. Marillac D. Hamani C.. (2018). Chronic deep brain stimulation in an Alzheimer’s disease mouse model enhances memory and reduces pathological hallmarks. Brain Stimul. 11, 435–444. doi: 10.1016/j.brs.2017.11.012, PMID: 29246746

[ref77] Marcantoni A. Raymond E. F. Carbone E. Marie H. (2014). Firing properties of entorhinal cortex neurons and early alterations in an Alzheimer’s disease transgenic model. Pflugers Arch. 466, 1437–1450. doi: 10.1007/s00424-013-1368-z, PMID: 24132829

[ref78] Martinez-Losa M. Tracy T. E. Ma K. Verret L. Clemente-Perez A. Khan A. S.. (2018). Nav1.1-overexpressing interneuron transplants restore brain rhythms and cognition in a mouse model of Alzheimer’s disease. Neuron 98, 75–89.e5. doi: 10.1016/j.neuron.2018.02.029, PMID: 29551491 PMC5886814

[ref79] Martorell A. J. Paulson A. L. Suk H. J. Abdurrob F. Drummond G. T. Guan W.. (2019). Multi-sensory gamma stimulation ameliorates Alzheimer’s-associated pathology and improves cognition. Cell 177, 256–271.e22. doi: 10.1016/j.cell.2019.02.014, PMID: 30879788 PMC6774262

[ref80] Mehak S. F. Shivakumar A. B. Kumari S. Muralidharan B. Gangadharan G. (2022). Theta and gamma oscillatory dynamics in mouse models of Alzheimer’s disease: a path to prospective therapeutic intervention. Neurosci. Biobehav. Rev. 136:104628. doi: 10.1016/j.neubiorev.2022.104628, PMID: 35331816

[ref81] Melzer S. Michael M. Caputi A. Eliava M. Fuchs E. C. Whittington M. A.. (2012). Long-range-projecting GABAergic neurons modulate inhibition in hippocampus and entorhinal cortex. Science 335, 1506–1510. doi: 10.1126/science.1217139 22442486

[ref82] Nakazono T. Lam T. N. Patel A. Y. Kitazawa M. Ph.D Saito T. Ph.D Saido T. C. Ph.D. (2017). Impaired in vivo gamma oscillations in the medial entorhinal cortex of Knock-in Alzheimer model. Front. Syst. Neurosci. 11:48. doi: 10.3389/fnsys.2017.00048, PMID: 28713250 PMC5491963

[ref83] Navarro Schröder T. Haak K. V. Zaragoza Jimenez N. I. Beckmann C. F. Doeller C. F. (2015). Functional topography of the human entorhinal cortex. eLife 4:e06738. doi: 10.7554/eLife.06738, PMID: 26052748 PMC4458840

[ref84] Nuriel T. Angulo S. L. Khan U. Ashok A. Chen Q. Figueroa H. Y.. (2017). Neuronal hyperactivity due to loss of inhibitory tone in APOE4 mice lacking Alzheimer’s disease-like pathology. Nat. Commun. 8:1464. doi: 10.1038/s41467-017-01444-0, PMID: 29133888 PMC5684208

[ref85] Palop J. J. Mucke L. (2016). Network abnormalities and interneuron dysfunction in Alzheimer disease. Nat. Rev. Neurosci. 17, 777–792. doi: 10.1038/nrn.2016.141, PMID: 27829687 PMC8162106

[ref86] Park M. Hoang G. M. Nguyen T. Lee E. Jung H. J. Choe Y.. (2021). Effects of transcranial ultrasound stimulation pulsed at 40 Hz on Aβ plaques and brain rhythms in 5×FAD mice. Transl. Neurodegener. 10:48. doi: 10.1186/s40035-021-00274-x, PMID: 34872618 PMC8650290

[ref87] Pennanen C. Kivipelto M. Tuomainen S. Hartikainen P. Hänninen T. Laakso M. P.. (2004). Hippocampus and entorhinal cortex in mild cognitive impairment and early AD. Neurobiol. Aging 25, 303–310. doi: 10.1016/S0197-4580(03)00084-8, PMID: 15123335

[ref88] Petrache A. L. Rajulawalla A. Shi A. Wetzel A. Saito T. Saido T. C.. (2019). ‘Aberrant excitatory-inhibitory synaptic mechanisms in entorhinal cortex microcircuits during the pathogenesis of Alzheimer’s disease. Cereb. Cortex 29, 1834–1850. doi: 10.1093/cercor/bhz016, PMID: 30766992 PMC6418384

[ref89] Pitkänen A. Pikkarainen M. Nurminen N. Ylinen A. (2000). Reciprocal connections between the amygdala and the hippocampal formation, perirhinal cortex, and postrhinal cortex in rat. A review. Ann. N. Y. Acad. Sci. 911, 369–391. doi: 10.1111/j.1749-6632.2000.tb06738.x 10911886

[ref90] Price J. L. Ko A. I. Wade M. J. Tsou S. K. McKeel D. W. Morris J. C. (2001). Neuron number in the entorhinal cortex and CA1 in preclinical Alzheimer disease. Arch. Neurol. 58, 1395–1402. doi: 10.1001/archneur.58.9.1395, PMID: 11559310

[ref91] Quilichini P. Sirota A. Buzsáki G. (2010). Intrinsic circuit organization and theta-gamma oscillation dynamics in the entorhinal cortex of the rat. J. Neurosci. Off. J. Soc. Neurosci. 30, 11128–11142. doi: 10.1523/JNEUROSCI.1327-10.2010, PMID: 20720120 PMC2937273

[ref92] Rodriguez G. A. Barrett G. M. Duff K. E. Hussaini S. A. (2020). Chemogenetic attenuation of neuronal activity in the entorhinal cortex reduces Aβ and tau pathology in the hippocampus. PLoS Biol. 18:e3000851. doi: 10.1371/journal.pbio.3000851, PMID: 32822389 PMC7467290

[ref93] Sahara N. Yanai R. (2023). Limitations of human tau-expressing mouse models and novel approaches of mouse modeling for tauopathy. Front. Neurosci. 17:1149761. doi: 10.3389/fnins.2023.1149761, PMID: 37152607 PMC10157230

[ref94] Salimi M. Tabasi F. Abdolsamadi M. Dehghan S. Dehdar K. Nazari M.. (2022). Disrupted connectivity in the olfactory bulb-entorhinal cortex-dorsal hippocampus circuit is associated with recognition memory deficit in Alzheimer’s disease model. Sci. Rep. 12:4394. doi: 10.1038/s41598-022-08528-y, PMID: 35292712 PMC8924156

[ref95] Save E. Sargolini F. (2017). Disentangling the role of the MEC and LEC in the processing of spatial and non-spatial information: contribution of lesion studies. Front. Syst. Neurosci. 11:81. doi: 10.3389/fnsys.2017.00081, PMID: 29163076 PMC5663729

[ref503] Schöll M. Lockhart S. N. Schonhaut D. R. Schwimmer H. D. Schöll M. Rabinovici G. D. Jagust W. J.. (2016). PET Imaging of Tau Deposition in the Aging Human Brain. Neuron 89:971982. doi: 10.1016/j.neuron.2016.01.028 PMC4779187 26938442

[ref96] Schultz M. K. Gentzel R. Usenovic M. Gretzula C. Ware C. Parmentier-Batteur S.. (2018). Pharmacogenetic neuronal stimulation increases human tau pathology and trans-synaptic spread of tau to distal brain regions in mice. Neurobiol. Dis. 118, 161–176. doi: 10.1016/j.nbd.2018.07.003, PMID: 30049665

[ref97] Stam C. J. van der Made Y. Pijnenburg Y. A. L. Scheltens P. (2003). EEG synchronization in mild cognitive impairment and Alzheimer’s disease. Acta Neurol. Scand. 108, 90–96. doi: 10.1034/j.1600-0404.2003.02067.x 12859284

[ref98] Stone S. S. D. Teixeira C. M. DeVito L. M. Zaslavsky K. Josselyn S. A. Lozano A. M.. (2011). Stimulation of entorhinal cortex promotes adult neurogenesis and facilitates spatial memory. J. Neurosci. Off. J. Soc. Neurosci. 31, 13469–13484. doi: 10.1523/JNEUROSCI.3100-11.2011, PMID: 21940440 PMC6623309

[ref99] Stranahan A. M. Mattson M. P. (2010). Selective vulnerability of neurons in layer II of the entorhinal cortex during aging and Alzheimer’s disease. Neural Plast. 2010:108190. doi: 10.1155/2010/108190, PMID: 21331296 PMC3039218

[ref100] Targa Dias Anastacio H. Matosin N. Ooi L. (2022). Neuronal hyperexcitability in Alzheimer’s disease: what are the drivers behind this aberrant phenotype? Transl. Psychiatry 12:257. doi: 10.1038/s41398-022-02024-7, PMID: 35732622 PMC9217953

[ref101] Thal D. R. Rüb U. Orantes M. Braak H. (2002). Phases of a beta-deposition in the human brain and its relevance for the development of AD. Neurology 58, 1791–1800. doi: 10.1212/wnl.58.12.1791, PMID: 12084879

[ref102] Thorns V. Licastro F. Masliah E. (2001). ‘Locally reduced levels of acidic FGF lead to decreased expression of 28-kda calbindin and contribute to the selective vulnerability of the neurons in the entorhinal cortex in Alzheimer’s disease. Neuropathology 21, 203–211. doi: 10.1046/j.1440-1789.2001.00399.x, PMID: 11666017

[ref103] Titiz A. S. Hill M. R. H. Mankin E. A. Aghajan Z. M. Eliashiv D. Tchemodanov N.. (2017). Theta-burst microstimulation in the human entorhinal area improves memory specificity. eLife 6:e29515. doi: 10.7554/eLife.29515 29063831 PMC5655155

[ref104] Tukker J. J. Beed P. Brecht M. Kempter R. Moser E. I. Schmitz D. (2022). Microcircuits for spatial coding in the medial entorhinal cortex. Physiol. Rev. 102, 653–688. doi: 10.1152/physrev.00042.2020, PMID: 34254836 PMC8759973

[ref105] Van Cauter T. Camon J. Alvernhe A. Elduayen C. Sargolini F. Save E. (2013). Distinct roles of medial and lateral entorhinal cortex in spatial cognition. Cerebral Cortex 23, 451–459. doi: 10.1093/cercor/bhs033 22357665

[ref106] Van Groen T. (2001). Entorhinal cortex of the mouse: cytoarchitectonical organization. Hippocampus 11, 397–407. doi: 10.1002/hipo.1054, PMID: 11530844

[ref107] Velayudhan L. Proitsi P. Westman E. Muehlboeck J. S. Mecocci P. Vellas B.. (2013). Entorhinal cortex thickness predicts cognitive decline in Alzheimer’s disease. J. Alzheimers Dis. 33, 755–766. doi: 10.3233/JAD-2012-121408 23047370

[ref108] Verret L. Mann E. O. Hang G. B. Barth A. M. I. Cobos I. Ho K.. (2012). Inhibitory interneuron deficit links altered network activity and cognitive dysfunction in Alzheimer model. Cell 149, 708–721. doi: 10.1016/j.cell.2012.02.046, PMID: 22541439 PMC3375906

[ref109] Vogels T. Leuzy A. Cicognola C. Ashton N. J. Smolek T. Novak M.. (2020). Propagation of tau pathology: integrating insights from postmortem and in vivo studies. Biol. Psychiatry 87, 808–818. doi: 10.1016/j.biopsych.2019.09.019, PMID: 31735253

[ref110] Vossel K. A. Beagle A. J. Rabinovici G. D. Shu H. Lee S. E. Naasan G.. (2013). Seizures and epileptiform activity in the early stages of Alzheimer disease. JAMA Neurol. 70, 1158–1166. doi: 10.1001/jamaneurol.2013.136, PMID: 23835471 PMC4013391

[ref111] Ward L. M. (2003). Synchronous neural oscillations and cognitive processes. Trends Cogn. Sci. 7, 553–559. doi: 10.1016/j.tics.2003.10.012 14643372

[ref112] Whitwell J. L. Przybelski S. A. Weigand S. D. Knopman D. S. Boeve B. F. Petersen R. C.. (2007). ‘3D maps from multiple MRI illustrate changing atrophy patterns as subjects progress from mild cognitive impairment to Alzheimer’s disease. Brain J. Neurol. 130, 1777–1786. doi: 10.1093/brain/awm112 PMC2752411 17533169

[ref113] Witter M. P. Doan T. P. Jacobsen B. Nilssen E. S. Ohara S. (2017). Architecture of the entorhinal cortex a review of entorhinal anatomy in rodents with some comparative notes. Front. Syst. Neurosci. 11:46. doi: 10.3389/fnsys.2017.00046, PMID: 28701931 PMC5488372

[ref114] Wu J. W. Hussaini S. A. Bastille I. M. Rodriguez G. A. Mrejeru A. Rilett K.. (2016). Neuronal activity enhances tau propagation and tau pathology in vivo. Nat. Neurosci. 19, 1085–1092. doi: 10.1038/nn.4328, PMID: 27322420 PMC4961585

[ref115] Xia F. Yiu A. Stone S. S. D. Oh S. Lozano A. M. Josselyn S. A.. (2017). Entorhinal cortical deep brain stimulation rescues memory deficits in both Young and old mice genetically engineered to model Alzheimer’s disease. Neuropsychopharmacology 42, 2493–2503. doi: 10.1038/npp.2017.100, PMID: 28540926 PMC5686482

[ref116] Xu W. Fitzgerald S. Nixon R. A. Levy E. Wilson D. A. (2015). Early hyperactivity in lateral entorhinal cortex is associated with elevated levels of AβPP metabolites in the Tg2576 mouse model of Alzheimer’s disease. Exp. Neurol. 264, 82–91. doi: 10.1016/j.expneurol.2014.12.008, PMID: 25500142 PMC4324092

[ref117] Yamada K. Holth J. K. Liao F. Stewart F. R. Mahan T. E. Jiang H.. (2014). Neuronal activity regulates extracellular tau in vivo. J. Exp. Med. 211, 387–393. doi: 10.1084/jem.20131685, PMID: 24534188 PMC3949564

[ref118] Ye J. Witter M. P. Moser M. B. Moser E. I. (2018). Entorhinal fast-spiking speed cells project to the hippocampus. Proc. Natl. Acad. Sci. USA 115, E1627–E1636. doi: 10.1073/pnas.1720855115, PMID: 29386397 PMC5816210

[ref119] Yeh C.-Y. Vadhwana B. Verkhratsky A. Rodríguez J. J. (2011). Early astrocytic atrophy in the entorhinal cortex of a triple transgenic animal model of Alzheimer’s disease. ASN Neuro 3, 271–279. doi: 10.1042/AN20110025, PMID: 22103264 PMC3243908

[ref120] Ying J. Keinath A. T. Lavoie R. Vigneault E. el Mestikawy S. Brandon M. P. (2022). Disruption of the grid cell network in a mouse model of early Alzheimer’s disease. Nat. Commun. 13:886. doi: 10.1038/s41467-022-28551-x, PMID: 35173173 PMC8850598

[ref121] Zott B. Hong W. Unger F. Chen-Engerer H. J. Frosch M. P. Sakmann B.. (2019). ‘A vicious cycle of β amyloid-dependent neuronal hyperactivation. Science 365, 559–565. doi: 10.1126/science.aay0198, PMID: 31395777 PMC6690382

